# Thermodynamic anomalies, polyamorphism and all that

**DOI:** 10.1098/rsta.2022.0336

**Published:** 2023-10-16

**Authors:** Domagoj Fijan, Mark Wilson

**Affiliations:** Department of Chemistry, Physical and Theoretical Chemistry Laboratory, University of Oxford, Oxford, UK

**Keywords:** thermodynamics, phase diagrams, polymorphism

## Abstract

The appearance and evolution of thermodynamics anomalies, and related properties, are studied for two classes of system, modelling those dominated by covalent and ionic interactions, respectively. Such anomalies are most familiar in the density but are also present in other thermodynamics variables such as the compressibility and heat capacity. By systematically varying key model parameters the emergence and evolution of these anomalies can be tracked across the phase space. The interaction of the anomalies can often be rationalized by thermodynamics ‘rules’. The emergence of these anomalies may also be correlated with the appearance of polyamorphism, the existence of multiple amorphous states which differ in density and entropy.

This article is part of the theme issue ‘Exploring the length scales, timescales and chemistry of challenging materials (Part 1)’.

## 1. Introduction

Water shows perhaps the most widely known thermodynamic anomaly, displaying a maximum in the density at $T∼4^{\circ }C$ (at ambient pressure). However, such anomalous behaviour is not limited to water. For example, anomalous behaviour is observed in systems ranging from silicon [1,2], germanium [3] and carbon, to ${\text{BeF}}_{2}$, ${\text{SiO}}_{2}$ and ${\text{GeO}}_{2}$ [1,4]. The detailed origin of such behaviour is subtle and often ‘buried’ in the balance of the dominant interatomic interactions. Si and Ge, for example, are classic ‘covalent’ materials, while ${\text{BeF}}_{2}$ and ${\text{SiO}}_{2}$ can be considered as dominated by ionic (electrostatic) interactions. Water effectively shows a mixture of covalent and ionic interactions, as well as significant hydrogen-bonding effects [4].

Computer simulation models offer potentially unique insight into anomalous behaviour as, not only are the location of all atoms known exactly, but the precise details of the interatomic interactions can be systematically controlled and varied. Many different potential models have been applied with both electrostatic potentials (either polarizable or non-polarizable [5–13]) and covalent models, such as the Stillinger–Weber (SW) potential, originally used to model silicon, [14] displaying anomalous behaviour in the liquid and/or supercooled region of the phase diagram. Furthermore, although anomalous behaviour is most well-known for the density, related behaviour is observed in properties such as the compressibility and heat capacity [7,12,15–48]. The manner in which these anomalies interact in the phase space is governed by the laws of thermodynamics. Significant previous work has focused on how the various anomalies (and the related stability limits (SL)) interact with each other [18,36,37,49–56]. In recent work, we have derived a more complete set of interaction rules [57].

The density maximum in ${\text{H}}_{2}O$ at $T∼4^{\circ }C$ is specific to ambient pressure. Determining the temperature at which the anomaly appears as a function of pressure allows a locus of the turning points to be constructed which is termed a temperature of maximum density (TMD) line. In the systems listed above these appear to arise from the subtle disruption of a basically tetrahedral network as a function of temperature and/or pressure.

In general, two basic modelling strategies have traditionally been employed. Firstly, relatively accurate models are used to model specific systems, for example, ${\text{SiO}}_{2}$ [58–61], ${\text{BeF}}_{2}$ [9,10,61], ${\text{GeO}}_{2}$ [61] and ${\text{H}}_{2}$O [15,17,18,52,61–64]. Secondly, more generic models are used, with examples including the SW [65,66], ramp [60,67–69] or core-softened [70] potentials. Recent work has highlighted how the location of critical points, focusing on both colloidal systems [71] and modified water potentials [72], depends on the detail of the underlying potential model.

Systems such as ${\text{SiO}}_{2}$ and ${\text{BeF}}_{2}$ are dominated by electrostatic interactions [73–76]. As a result, anions surround cations with the nearest-neighbour coordination number (the short-range order). This coordination is effectively controlled by the relative ion sizes (usually in terms of ‘radius-ratio rules’). ${\text{SiO}}_{2}$, ${\text{GeO}}_{2}$ and ${\text{BeF}}_{2}$ show structural similarities in the sense that the nearest-neighbour coordination polyhedra are ${\text{MX}}_{4}$ tetrahedra. However, their ordering beyond the short-range may differ as the bridging bond angles between neighbouring tetrahedra vary from ${\bar{\theta }}_{\mathrm{MXM}}≃130^{\circ }$ for ${\text{GeO}}_{2}$, to ${\bar{\theta }}_{\mathrm{MXM}}≃145^{\circ }$ for ${\text{SiO}}_{2}$, to ${\bar{\theta }}_{\mathrm{MXM}}≃155^{\circ }$ for ${\text{BeF}}_{2}$ [77]. The M–X–M bond angles are controlled by many-body (ion polarization) interactions [78,79]. Dipoles induced on the bridging anions act to introduce negative charge between neighbouring cation centres, effectively screening the cation–cation repulsive electrostatic interaction [80]. The magnitude of these dipoles depends on the magnitude of the local electric field and the anion dipole polarizability [78,79,81] and effectively controls the system topology. As a result, in an ionic model these bond angles can be controlled by a single model parameter, the (anion) dipole polarizability, α. This means that generic models can be applied in which α is systematically varied [81,82]. For the ${\text{MX}}_{2}$ stoichiometry polarizable-ion models (PIMs) have been developed for ${\text{GeSe}}_{2}$ [60], ${\text{GeO}}_{2}$ [83], ${\text{ZnCl}}_{2}$ [60,84] and ${\text{SiO}}_{2}$ [85].

Systems such as silicon and water show highly complex crystalline phase diagrams which show complex changes in coordination environment on different length scales. In the disordered state silicon shows low- and high-density amorphous forms (LDA and HDA) which are characterized by showing different densities and entropies, and which display a change in (electronic) conductivity [86]. More recent experiments and simulations highlight how silicon may be more ‘water-like’, displaying additional amorphous forms [87,88].

In this paper, two classes of model will be applied to consider systems dominated by covalent and ionic interactions, respectively. Specific systems, namely Si and ${\text{BeF}}_{2}$, will be considered, as well as the effect of systematically varying a key parameter which controls each respective potential model.

## 2. Models and methods

### (a) Potential models

In the SW potential [14], the energy, U, is expressed as

2.1 $$\begin{array}{rl} & U=\sum_{i}\sum_{j>i}U_{2}(r_{ij})+\sum_{i}\sum_{j>i}\sum_{k>j}U_{3}(r_{ij},r_{ik},\theta_{ijk})\\ & U_{2}(r_{ij})=A\left[B{\left(\frac{1}{r_{ij}}\right)}^{p}-{\left(\frac{1}{r_{ij}}\right)}^{q}\right]{\text{e}}^{1/(r_{ij}-a)}\\ \;\mathrm{and}\; & U_{3}(r_{ij},r_{ik},\theta_{ijk})=\lambda {\left[\cos\theta_{ijk}-\cos\theta_{0}\right]}^{2}\;{\text{e}}^{\gamma /\left(r_{ij}-a\right)}\;{\text{e}}^{\gamma /\left(r_{ik}-a\right)}. \end{array}\}$$

The atomic interactions are, therefore, controlled by the parameter set $\left\{A,\theta_{0},B,p,q,a,\lambda ,\gamma \right\}$. Note that the first term ($U_{2}$) is a pair potential and would be equivalent to a Lennard–Jones form for p=12 and q=6, modified by an exponential truncation term which effectively removes the high separation dispersive interations, truncating the pair potential on a length-scale controlled by the parameter a. The second term ($U_{3}$) is a three body term which contains the angle between the three bonded atoms labelled ijk. As a result, the overall potential energy is essentially of the form $U=U_{2}+\lambda U_{3}$ and so the parameter λ controls the relative magnitude of the two- and three-body terms. Previous work shows that values of λ=17–28 show a density anomaly [46,89]. Furthermore, varying λ has been found to model chemically related systems, notably silicon (λ=21 [14]), phosphorus (λ≃16.5 [90,91]), germanium (λ=20 [92]), carbon (λ=26.2 [93]) and even water (λ=23.15 [94]).

For the ionic systems, the potential model used is as described in [8]. Short-range interatomic interactions are accounted for by a modified Born–Mayer [95] potential (see [96] and references therein),

2.2 $$U_{sr}(r_{ij})=B_{ij}\;e^{-a_{ij}r_{ij}}-\sum_{n=6,8,10\cdots }\frac{C_{n}^{ij}}{r_{ij}^{n}}f_{n}(r_{ij}),$$

where $B_{ij}$ and $a_{ij}$ control the ion radii and the rate of decay of the repulsive wall, respectively, $C_{n}^{ij}$ and $f_{n}(r_{ij})$ are the dispersion coeficcients and respective damping functions [97]) of the form suggested by Tang & Toennies [98]. Full ionic charges (${\text{Be}}^{2+}$, ${\text{F}}^{-}$) are used throughout.

The pair potential is augmented by a description of ion polarization, introducing a many-body character and modelled using a PIM [80]. In the PIM, the dipole polarizability, α, and a short-range damping parameter (SRDP) control the magnitude of the induced dipoles [99–101]. The dipole polarizabilities and SRDPs can be obtained from *ab initio* electronic structure calculations [99–103]. Here a model for ${\text{BeF}}_{2}$ is employed which was parameterized by reference to density-functional calculations [8,104]. The full potential parameter set is given in [104]. The anion polarizability, $\alpha_{F^{-}}$, in ${\text{BeF}}_{2}$ is $\alpha_{F^{-}}=7.09$ au [8], smaller than the free ion value of $\alpha_{F^{-}}^{\text{FREE}}∼16.8\;\text{au}$ [105] as the anion is compressed in the condensed environment [99]. The polarizability of the ${\text{Be}}^{2+}$ cation is vanishingly small ($\alpha_{{\text{Be}}^{2+}}=0.052$ au [106]) and sits at the centre of a tetrahedron of anions whose symmetry precludes the formation of electric fields at the cation site. As a result, cation polarization can be neglected.

As noted in the Introduction, the topology of the network (i.e. the order beyond the short-range tetrahedral order) is controlled by the anion polarizability. As a result, it is potentially insightful to consider variations of the ${\text{BeF}}_{2}$ model in which the anion polarizability itself is systematically varied between zero (corresponding to a rigid-ion model—RIM) and the full value of $\alpha_{F^{-}}=7.09$ au (corresponding to the full PIM). In this work, we employ values of 0, 1, 2, 3, 4, 5, 6 and 7.09 au.

### (b) Methods

To model the covalent systems we employ molecular dynamics (MD) on systems containing 500 atoms, in the NVT ensemble using LAMMPS [107] with the temperature maintained using Nosé–Hoover thermostats [108,109]. Isochores are obtained over a range of volumes and temperatures within the stability limit of the potential model as discussed in [110]. Typically systems are equilibrated for t∼2 ns with production runs of t∼2.5 ns. We analyse the thermodynamic state space adjacent to the liquid–vapour stability limit using longer production runs of t∼150 ns and with increased temperature and volume sampling [2]. At state points for which dynamical arrest was observed on the simulation time-scale replica exchange molecular dynamics (REMD) [111,112] were employed to enable a more complete exploration of the disordered state configurational space and as described in [110]. The thermodynamic data are analysed using the NumPy and SciPy packages [113] and the anomalies and related properties are identified as described in [110]. Note that the liquid–vapour stability limits are identified by isolating the lowest pressure point on an isotherm for each temperature which gives an upper bound estimate of the liquid–vapour spinodal line. As a result this line is referred to as the ‘stability limit’ (SL) rather than the ‘spinodal’.

The location of any liquid–liquid critical points (LLCPs) are estimated from the liquid–liquid spinodals, calculated by identifying pairs of minima and maxima on the isotherms. It should be noted that the presence of such critical points remains controversial. For example, free energy studies indicate that the LLCP locations were previously reported as unstable with respect to a crystalline phase [114]. Our results agree as we are unable to obtain a stable equilibrium in the relevant region of the phase space. The unequilibrated data indicates the existence of two LLCPs in this unstable region, consistent with the concept of ‘virtual criticality’ suggested for water [115]. The existence (or not) of multiple LLCPs does not affect the general discussions presented here.

For the ionic models MD simulations are performed in the NVT ensemble with a system of 576 ${\text{MX}}_{2}$ molecules with constant temperature again maintained using Nosé–Hoover thermostats [108,109]. Production runs were on a time-scale of t≈1 ns for the RIM and t≈360 ps for the PIM, a reflection that a typical PIM simulation requires a computational effort of around an order of magnitude more than the RIM counterpart as a result of evaluating the many-body interactions. The thermodynamic data are extracted and analysed as for the covalent model.

## 3. Summary of anomalous behaviour

A thermodynamic analysis can establish the constraints to describe how the anomaly loci interact. In addition, the analogous analysis indicates how such anomalies interact with related properties such as critical points and stability limits (see, for example, [57] and references therein). The conditions for interaction are obtained directly from the underlying thermodynamic relations using standard manipulations and, as a result, are exact. For clarity, the temperatures of the maxima in the respective properties are referred to as temperature of maximum density (TMD), maximum compressibility (TMC) and maximum heat capacity (TMH), respectively, while the corresponding minima are termed temperature of minimum density, compressibility and heat capacity (TminD, TminC and TminH), respectively.

The key interactions are summarized here:

— Density and compressibility anomaly loci (i.e. TMX or TminX, X={C,D}) ‘transform’ from a minimum to a maximum when ${\left(\partial p/\partial T\right)}_{\text{TMX or TminX}}=0$ in the pT projection. The heat capacity shows the analogous change at ${\left(\partial p/\partial T\right)}_{\text{TMH or TminH}}=\infty$.
— Although the density anomalies are *not required* to collide with the stability limit, if such a collision occurs then the gradient (${\left(\text{d}p/\text{d}T\right)}_{\text{SL}}$) of the stability limit locus at the collision state point is zero, in the pT projection [57].
— A compressibility anomaly may intersect a density anomaly when the latter has an infinite gradient (${\left(\text{d}p/\text{d}T\right)}_{\text{TMD, TminD}}=\infty$) in the pT projection [52].
— Heat capacity anomalies intersect density anomalies when the latter have zero gradient (${\left(\text{d}p/\text{d}T\right)}_{\text{TMD, TminD}}=0$) in the pT projection.
— A heat capacity anomaly locus with zero gradient can only be intersected with an infinite gradient compressibility anomaly.

Regarding the density and compressibility anomalies, the singularity-free interpretation suggests three alternative scenarios for their respective behaviour [52]. The three possibilities differ in the respective relationship of the pressure and temperature at which the TMC and TminC loci merge compared to the point at which one crosses the density anomaly. A compressibility anomaly locus intercepting the TMD at a positive TminC gradient is termed TEC1, intersection with a negative gradient is termed TEC3, and a simultaneous TMC/TminC and intersection with the TMD is termed TEC2.

## 4. Results

The results are divided into those for the specific models for Si and ${\text{BeF}}_{2}$, then extended to more generic models in which the key parameters identified above are systematically varied.

### (a) Specific systems: Si and BeF_2_

**Figure 1.**
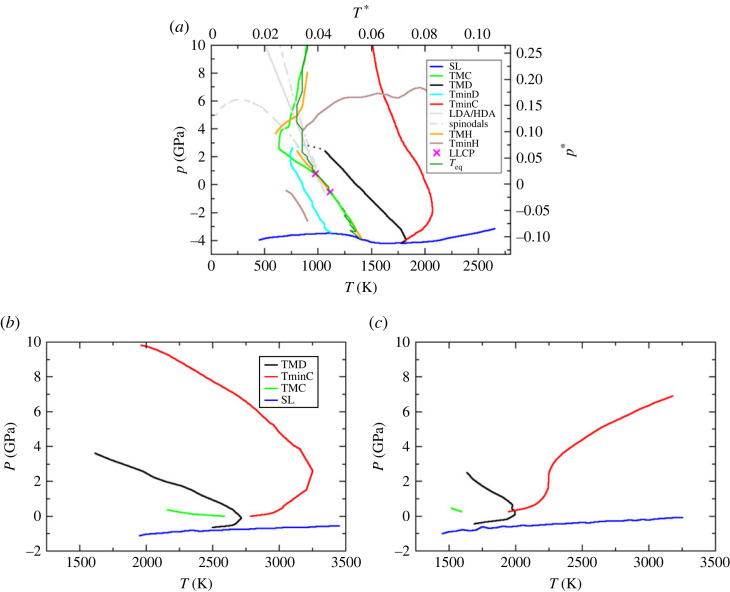
The locations of key thermodynamics anomaly loci and related properties for (*a*) a SW potential appropriate to model Si (λ=21), and (*b*,*c*) an ionic model for BeF_2_ in which the anion polarizability is set to zero ((*b*), corresponding to a RIM) and in which α=5.0 au (*c*). All are shown in the pT plane. In (*a*,*b*), the identities of the specific lines and points are indicated by the legends. In (*a*), the dashed cyan and black lines show the extrapolation of the TminD and TMD loci at high pressure. The identities of the lines in (*c*) are equivalent to those in (*b*). (Online version in colour.)

Figure 1*a* shows the locations of a range of thermodynamic anomalies and related properties, obtained using a SW potential parameterized to model silicon.^1^ The TMD locus ‘transforms’ to a TminD at high pressure (p∼3 GPa) but does not do so at the low pressure ‘limit’ as both the TMD and TminD loci intersect the stability limit locus. The high pressure crossover is difficult to observe directly owing to the inherent large fluctuations. The figure shows the extrapolated curves in this region as a guide to the eye. The gradient of the SL locus in the pT plane is zero at these two intersections, as is required. In the absence of these interactions the TMD/TminD loci would form a closed loop. The TMD locus is re-entrant with a maximum temperature of T∼1800 K. The TminC and TMC loci intersect the TMD and TminD loci, respectively, where the density loci have infinite gradient, as required. The compressibility locus intersects the TMD with a positive gradient (equivalent to the TEC1 scenario in the singularity-free interpretation).

Figure 1*a* also highlights the identified pair of virtual LLCPs with the TMH locus emerging as required. The heat capacity loci intersect the density anomalies when the latter has zero gradient, again as required and corresponding to the respective TMD↔TminD crossovers. The TMH locus also transforms to the TminH near the high pressure TMD to TminD crossover. In addition, at high pressure and temperature the TminC and TminH lines intersect with near-orthogonal gradients. Both LLCPs and part of the TMH locus that transforms into TminH are located in the area of the phase diagram where we could not obtain proper equilibrium results. The temperature below which full equilibration is not possible is indicated as $T_{\text{eq}}$ in figure 1. $T_{\text{eq}}$ is determined by observing the arrest in the decay of the time correlation function extracted from the local tetrahedrality (see [110]). The results presented are an attempt to extend the equations of state into unstable parts of the phase diagram in order to obtain a more complete picture of behaviour of thermodynamic anomalies.

Figure 1*a* also shows the LDA/HDA coexistence curve (taken from [86]) along with the two associated spinodals. The coexistence line emerges from an LLCP (shown here as originally determined but close to the two current virtual critical points) and appears to go through the density anomaly locus loop at the higher pressure ‘transformation’ between the TMD and TminD. At higher pressures both the compressibility and heat capacity maxima appear to cross the LDA/HDA coexistence curve. It should be noted, however, that spinodals emanating from an amorphous–amorphous critical point do not interact with the anomalies determined here. The reason for this is that the extracted anomalies ‘live’ on the free energy surface of disordered states spanned by all structural (positional) degrees of freedom. The amorphous phases live on a subset of this surface (a subspace) defined by a limited set of configurational variables accessible inside this amorphous phase. This is due to the fact that ergodicity is hindered globally in both cases, for the amorphous phase and for parts of the phase diagram in the supercooled liquid state. However, the amorphous state is just a part of the overall disordered states free energy surface that is again locally hindered from exploring the whole disordered free energy surface.

Figure 1 shows a limited range of thermodynamic anomalies obtained for the ${\text{BeF}}_{2}$ RIM(i.e. α=0—(*b*)) and the same model with α=5 au (*c*). In both cases the TMD locus avoids intersection with the stability limit (and hence the SL gradient does not become zero). Again, the intersection of the respective density and compressibility anomalies occurs at the infinitely sloped part of the TMD. The TMC ↔ TminC crossover occurs at a temperature just below the TMD locus for the RIM (corresponding to scenario TEC-I). For the higher polarizability model the crossover shifts to lower temperature. The range of the thermodynamic phase space in which anomalies are observed appears narrower for the electrostatic models than for the covalent models. The effective observation of these anomalies requires the deeply supercooled regime to be traversed. The longer range interactions in the ionic systems mean that suppressing crystallization in these models is more difficult than for the covalent models.

**Figure 2.**
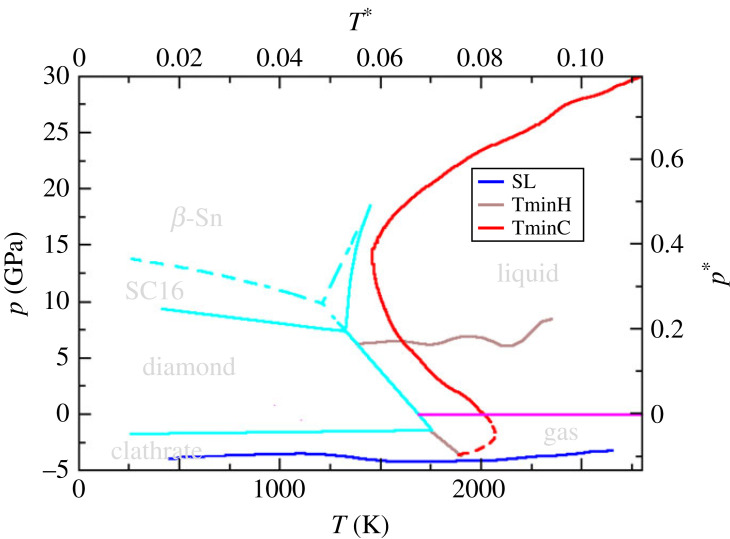
The thermodynamic anomalies observed for the silicon SW model (λ=21) in the fully equilibrated region of the pT phase space. Anomalies in the compressibility and heat capacity, as well as the stability limit, are shown as indicated by the legend. The figure also highlights the underlying crystal/liquid/gas phase diagram [116]. The liquid/crystal and crystal/crystal phase boundaries are shown in cyan with the dashed lines indicating metastable extensions to include the β-Sn polymorph. The magenta line shows the liquid/gas coexistence curve with the dashed red line highlighting the metastable extension of the TminC locus. (Online version in colour.)

Figure 2 highlights the range over which the anomalies are observed at strict thermodynamic equilibrium (i.e. not in the supercooled regime). The figure also shows the underlying crystalline phase diagram (from [116,117]). The phase diagram is shown on an expanded pressure scale compared to figure 1, which highlights the behaviour of the compressibility anomaly locus at high pressure, which shows a characteristic ‘S’ shape, which appears correlated with the liquid/crystal coexistence curves [89]. As a result, the ‘S’ shape arises from the combination of the underlying low pressure diamond crystal/liquid coexistence curve (which shows a negative Clapeyron slope) and the higher pressure SC16 crystal/liquid curve (positive Clapeyron slope).

### (b) General picture

**Figure 3.**
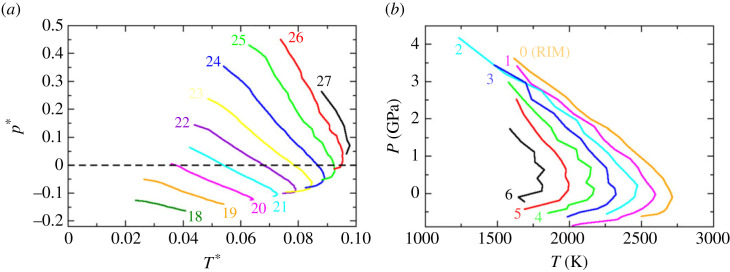
The evolution of the TMD loci for the (*a*) SW model as a function of the parameter λ and (*b*) the BeF_2_ model as a function of the anion polarizability, α. In both cases, the respective values of λ and α (the latter in atomic units) are indicated directly on the figures. The SW model data are shown as a function of the reduced pressure and temperature. (Online version in colour.)

As discussed in §2, a key rationale behind employing relatively simple potential models is the ability to modify the parameters in order to systematically explore the underlying parameter space and hence the fundamental interatomic interactions. In the present work, the structures in our two basic covalent and ionic models are controlled by the parameters λ and α, respectively. For example, figure 3 shows the change in the respective TMD loci for (*a*) the SW potential as a function of λ, and (*b*) the PIM for ${\text{BeF}}_{2}$ as a function of α. Recall that the parameter λ controls the relative magnitude of the two- and three-body interactions, while the parameter α controls the spatial relationship between nearest-neighbour tetrahedra. As λ increases the TMD shifts to higher pressure and temperature corresponding to an increase in the strength of the interatomic interactions. For the lowest value of λ for which a TMD can be detected (λ=18) the locus appears deep in the supercooled regime and at significant negative pressure and hence only a fragment of the locus appears accessible. Furthermore, the observed TMD is relatively weak in the sense that the respective density maximum has a relatively low curvature. For λ∼20 the TMD locus becomes re-entrant, a property which is retained up to the highest value for which a TMD is observed (λ=27). Figure 3*b* shows the analogous behaviour for the ${\text{BeF}}_{2}$ model as the anion polarizability, α, is increased. The TMD remains re-entrant at all values of α for which it is observed. As α increases the TMD retracts to lower temperature and becomes weaker and cannot be observed for the full anion polarizability.

**Figure 4.**
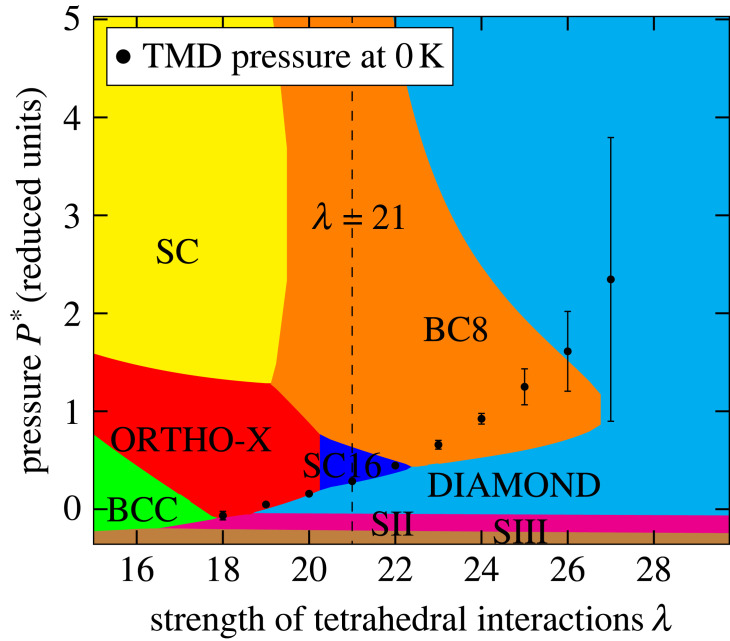
Phase diagram (determined at T=0 K) for the SW potential as a function of the parameter λ. The most stable crystal polymorph is indicated at each $\left\{\lambda ,p^{*}\right\}$ pair. SC refers to a simple cubic phase while SII and SIII are clathrate phases. The black circles represent the pressure limits of the respective TMD loci extracted to zero temperature. (Online version in colour.)

To further highlight the relationships between the appearance of the thermodynamic anomalies and the underlying phase diagram, figure 4 shows the T=0 K phase diagram as a function of pressure and the interaction parameter, λ. The phase diagram is constructed by determining the energy as a function of volume for each possible crystal polymorph, with the pressure obtained from the respective derivatives, and allowing the most favourable free energy to be identified at each state point. The figure also shows the pressures obtained by extending the TMD loci (using a simple linear fit) to T=0 K. The TMD is only detectable for values of λ for which both low- (tetrahedron-based) and high- (closer-packed) density can be stabilized with pressure. At λ=21, for example, which corresponds to the Si model discussed above, the diamond and SC16 crystal polymorphs are stable (and which correlate with the characteristic ‘S’-shape in the compressibility locus as discussed above).

## 5. Discussion

**Figure 5.**
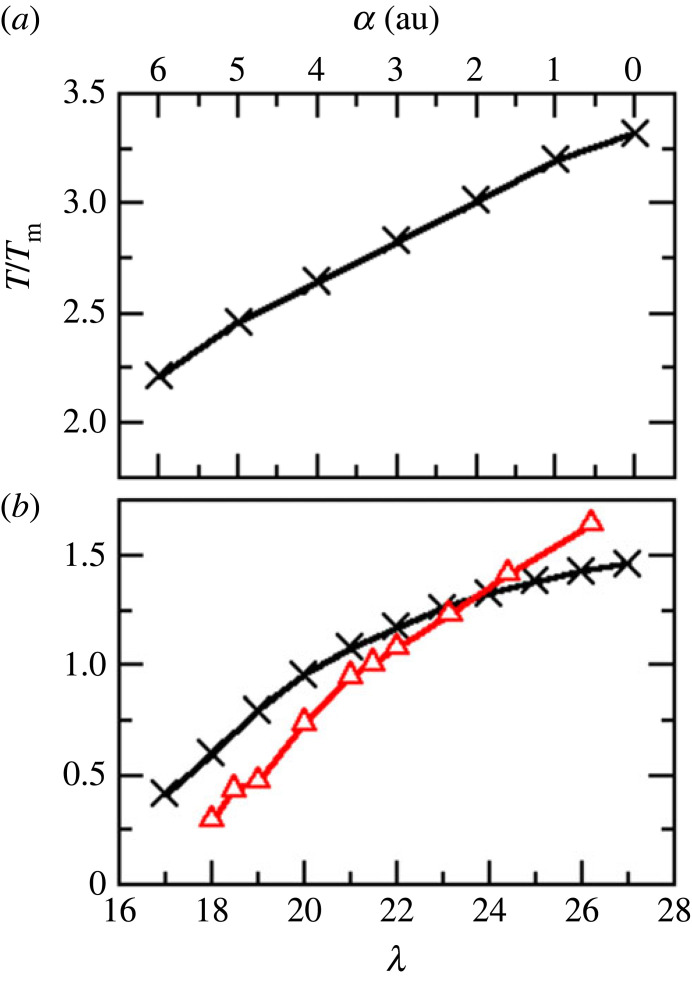
The variation of the highest temperature obtained by the TMD locus (×, black lines) for the ionic model as function of the anion polarizability, α, (*a*), and as a function of the Stillinger–Weber parameter λ for the covalent model (*b*). In (*b*), the liquid/crystal coexistence curve from [65] is also shown (red circles). (Online version in colour.)

Figure 5 shows the evolution of the maximum TMD temperature attained as a function of both λ (for the covalent model) and α (for the ionic model). An increase in λ in the covalent systems appears to have a similar effect to reducing α in the ionic models as both parameters effectively control the energetic balance between more open and more closed (low and high density) local environments. For the ${\text{BeF}}_{2}$ model employed here the TMD appears well above the likely melting point (i.e. $T/T_{m}>1$). However, for the SW potential the TMD lies entirely below the liquid/crystal coexistence curve (which is shown in figure 5 taken from [65]) for λ≲24 and partially emerges from beneath the melting curve for larger values of λ.

**Figure 6.**
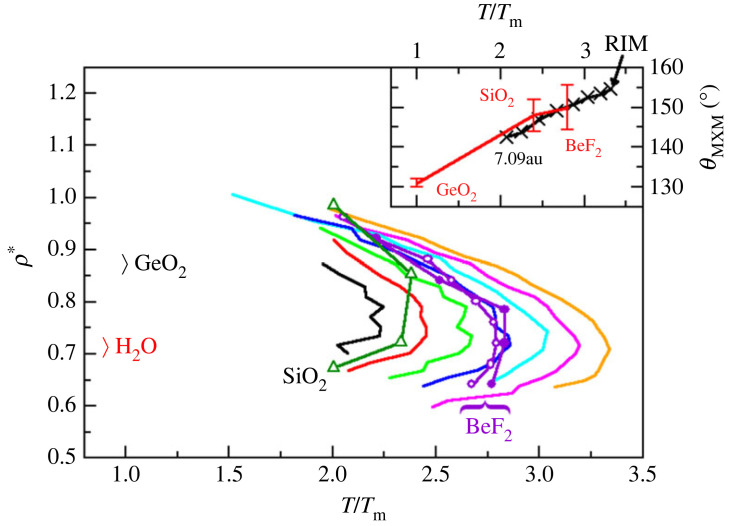
Temperature of maximum density loci obtained for the current BeF_2_ model (shown at seven values of the dipole polarizability—colours as for figure 3) and two previous models for BeF_2_ (closed circle from [61] and open circle from [118]), and models for SiO_2_, GeO_2_ and H_2_O (from [61]) as indicated directly on the figure. In all cases, the abscissa is reduced by the respective melting points and the ordinate is shown as a reduced density. (Online version in colour.)

Figure 6 shows the TMDs obtained for the ${\text{BeF}}_{2}$ model compared to those obtained for the chemically related systems ${\text{SiO}}_{2}$ and ${\text{GeO}}_{2}$ [61], along with anomalies for ${\text{BeF}}_{2}$ obtained using a different pair potential [61,118]. Analogous data for ${\text{H}}_{2}$O is shown for completeness [61]. In each case, the temperature and densities are shown in their respective reduced forms. The temperatures are rescaled using the respective melting points ($T_{m}=813$ K, 1996 K, 1389 K and 273 K for ${\text{BeF}}_{2}$, ${\text{SiO}}_{2}$, ${\text{GeO}}_{2}$ and ${\text{H}}_{2}\text{O}$, respectively—see the references given in [61]) and the number densities are rescaled such that $\rho^{*}=n_{0}\sigma^{3}$. The highest temperature reached by the TMD falls from ${\text{BeF}}_{2}⟶{\text{SiO}}_{2}⟶{\text{GeO}}_{2}⟶{\text{H}}_{2}\text{O}$. The inset to the figure shows the mean M–X–M bond angles, ${\bar{\theta }}_{\text{MXM}}$, that is, the bond angles between neighbouring ${\text{MX}}_{4}$ tetrahedra for the ${\text{BeF}}_{2}$ model over the full range of polarizabilities studied along with experimental values for ${\text{BeF}}_{2}$, ${\text{SiO}}_{2}$ and ${\text{GeO}}_{2}$ [77]. There is a clear correlation between the temperature scale at which the TMD locus emerges and the mean inter-tetrahedral bond angle, again as this bond angle is an effective measure of the energetic balance between local high and low density environments.

It is worth emphasizing that the use of relatively simple potential models, in which key interactions are readily identifiable and hence can be manipulated, is central to ‘unpicking’ the factors which control the emergence of the thermodynamic anomalies. In the present work, clear links can be drawn with existing experimental observations (for example, in silicon). It is likely, however, that such anomalies may be observed in more complex systems (for example, in mixtures). A systematic experimental search would be prohibitive but potential model investigations may be more realistic.

## 6. Conclusion

In this paper the appearance and evolution in the phase space of thermodynamic anomalies (and related properties) have been studied for two extreme classes of potential models, dominated by covalent and ionic interactions, respectively. In both cases, the potential models have been systematically modified by changing a single key parameter. This simple approach allows the anomalies to be followed to well into the supercooled regime and allows the complex interactions between them to be verified.

## Data Availability

This article has no additional data.

## Footnotes

1 Here, and in subsequent figures, temperatures and pressures are also shown in standard SW reduced units for which$1T^{*}=25\;157$ K and $1p^{*}=36.6$ GPa.
